# Epidemiological profile of multidrug-resistant and extensively drug-resistant *Mycobacterium Tubrculosis* among Congolese patients

**DOI:** 10.1186/s12941-021-00488-x

**Published:** 2021-12-17

**Authors:** Darrel Ornelle Elion Assiana, Jabar Babatunde Pacôme Achimi Abdul, Laure Stella Ghoma Linguissi, Micheska Epola, Jeannhey Christevy Vouvoungui, Albert Mabiala, Christopher Mebiame Biyogho, Jean Ronald Edoa, Bayodé Roméo Adegbite, Ayola Akim Adegnika, Linzy Elton, Julio Ortiz Canseco, Timothy D. McHugh, Gabriel Ahombo, Francine Ntoumi

**Affiliations:** 1 Fondation Congolaise pour la Recherche Médicale, Villa D6, Campus OMS, Djoué, Brazzaville, Republic of Congo; 2 Faculté des Sciences et Techniques, Université Marien Ngouabi, Brazzaville, Republic of Congo; 3 Centre de Recherches Médicales de Lambaréné, Lambaréné, Gabon; 4 Institut National de Recherche en Sciences de La Santé, Brazzaville, Republic of Congo; 5 Institute for Tropical Medicine, University of Tübingen, Tübingen, Germany; 6 German Center for Infection Research (DZIF), Tübingen, Germany; 7 Center for Clinical Microbiology, Division of Infection and Immunity, University College London, London, UK; 8 Service des Maladies Infectieuses, Hôpital de Réference de Makélékélé, Brazzaville, Republic of Congo

**Keywords:** Epidemiological profile, Multidrug-resistant TB, Extensively drug-resistant TB, Xpert MTB / RIF, Line Probe Assay SL, Republic of Congo

## Abstract

**Background:**

There is paucity of data on the prevalence and distribution of multidrug- Resistant-Tuberculosis (MDR-TB) in the Republic of Congo. Among the challenges resides the implementation of a robust TB resistance diagnostic program using molecular tools. In resource limited settings there is a need to gather data to enable prioritization of actions. The objective of this study was is to implement molecular tools as a best of diagnosing MDR and XDR-TB among presumptive tuberculosis patients referred to reference hospital of Makelekele in Brazzaville, Republic of the Congo.

**Methods:**

We have conducted a cross-sectional study, including a total of 92 presumptive pulmonary tuberculosis patients and who had never received treatment recruited at the reference hospital of Makelekele from October 2018 to October 2019. The socio-demographic and clinical data were collected as well as sputum samples. Rifampicin resistance was investigated using Xpert (Cepheid) and second-line TB drugs Susceptibility testing were performed by the Brucker HAIN Line Probe Assay (GenoType MTBDRsl VER 2.0 assay) method.

**Results:**

From the 92 recruited patients, 57 (62%) were found positive for the Mycobacterium tuberculosis complex. The prevalence of rifampicin-resistant tuberculosis (RR-TB) was 9.8% (9/92) and importantly 2.2% were pre-XDR/XDR.

**Conclusion:**

This study showed a high rate of rifampicin resistance and the presence of extensively drug-resistant tuberculosis in the study area in new patients. This study highlights the need for further studies of TB drug resistance in the country.

## Introduction

Tuberculosis (TB) is a major public health problem worldwide. The World Health Organization (WHO) estimated that there were 9,9 million new people of tuberculosis in 2020, of which 784,000 occurred among PLHIV and 157, 9000 people were found to have rifampicin resistant tuberculosis (RR-TB) [1]

For several decades, the emergence of MDR-TB as well as XDR-TB has been an obstacle to the control of the disease [2, 3]. Early detection of drug resistance is crucial to prevent transmission of drug-resistant TB and avoid mortality [4]. In low and middle income countries, the implementation of robust TB resistance diagnostic programs using molecular tools remains a challenge.

In the Republic of Congo, the annual incidence of tuberculosis in 2020 was estimated at 379 cases per 100,000 population and the proportion of co-infection HIV and tuberculosis was 112 cases per 100,000 population [5]. It is reported that MDR-TB accounts for 2.4% of new cases [6]. However, no national survey on MTB drug resistance has been carried out [7] and there is paucity of data on the prevalence and distribution of MDR-TB in the country. Such information is essential to facilitate effective control measures and the results of these tests are essential for clinicians in the design of the treatment regimen in the management of MDR-TB patients [3]. Failure to diagnose and treat MDR-TB patients have a negative impact on resistance level, transmission and mortality [3, 8].

The gold standard methods for the detection of MDR-TB includes in vitro culture and drug susceptibility testing (DST). These methods are time consuming, expensive [9] and also require a high-level biosafety facilities (Biosafety Level 3 laboratory) with qualified personnel [9]. Since several years, rapid molecular diagnostic tests have been developed to overcome the absence of in vitro culture capabilities like such as the Xpert MTB / RIF (Cepheid, Sunnyvale, CA, USA) system and Line probe Assays (LPA) with a focus on the rapid detection of TB drug resistance [10, 11]. Indeed, since 2010, WHO approved the Xpert MTB / Rifampicin test (Cepheid, Sunnyvale, CA, United States) as a routine tool to be used for screening suspected MDR-TB patients or TB-HIV co-infected individuals [12]. Furthermore, in 2016, WHO approved the use of version 2 of the HAIN GenoType MTBDRsl as the genotypic test for drug susceptibility testing to detect resistance to fluoroquinolones and injectable second-line drug (SLI) [13].

In 2013, The first GeneXpert was introduced in the Republic of Congo [14] but did not help to meet the expectations because of limited financial resources which caused shortages in cartridges. Under the support of the Central Africa clinical Research Network (CANTAM, www.cantam.org), the present study was carried out to provide reliable data on MDR- and XDR-TB profile among patients suspicious of pulmonary tuberculosis and consulting at the reference hospital of Makelekele in Southern area of Brazzaville, Republic of the Congo.

## Materials and methods

### Ethical approval and consent

The protocol of this study was submitted to the institutional ethics committee of the Fondation Congolaise pour la Recherche Médicale and ethical clearance released under the reference 015/CIE/FCRM/May 30, 2018. All participants aged ≥ 18 years old gave their written informed consent and < 18 years old patients provided an assent, in addition to the written informed consent from the parent or guardian. Confidentiality of data was ensured, prior inclusion into the study.

### Study location

The study was conducted in Brazzaville which is the political and administrative capital of the Republic of Congo. Study participants were enrolled at the Reference Hospital of Makelekele, which is the second largest hospital of reference located in the south of Brazzaville, and covers the first sanitary district of the city covering an estimated population size of 74,815 inhabitants and an area of 15.53 km^2^.

At Reference Hospital of Makelekele, the study was conducted in the infectious diseases department. All Presumptive tuberculosis individuals coming for consultation at Reference Hospital of Makelekele are welcomed in the infectious diseases Department. In 2018, the prevalence of TB patients confirmed by bacteriological tests was estimated at 17%, according to the hospital's registry.

### Type and population of study

This is a cross-sectional study which focus on the resistance pattern of circulating TB-strains in Brazzaville conducted at from October 2018 to October 2019 and targeted presumptive pulmonary tuberculosis participants and who had never received treatment recruited. Eligible participants were between 8 and 70 years of age, presenting TB clinical signs, without prior anti-TB treatment, voluntarily consented and assented to HIV testing, and residing in Brazzaville during the study period. Participants with diseases such as cancer, advanced HIV-AIDS, severe malaria and extra-pulmonary TB were excluded. The study was based only on pulmonary TB and all participants with extra-pulmonary TB were not eligible.

### Operational definitions

#### Presumptive tuberculosis participants

individuals with evocative symptoms of TB (coughing for 3 weeks or more, persistent and productive, sputum sometimes streaked with blood, chest pain, weight loss, tiredness, anorexia, fever and night sweats).

#### Acute cough

Cough as lasting less than three weeks.

#### Chronic cough

Cough lasting more than eight weeks.

### Sample collection and study design

**Fig. 1:**
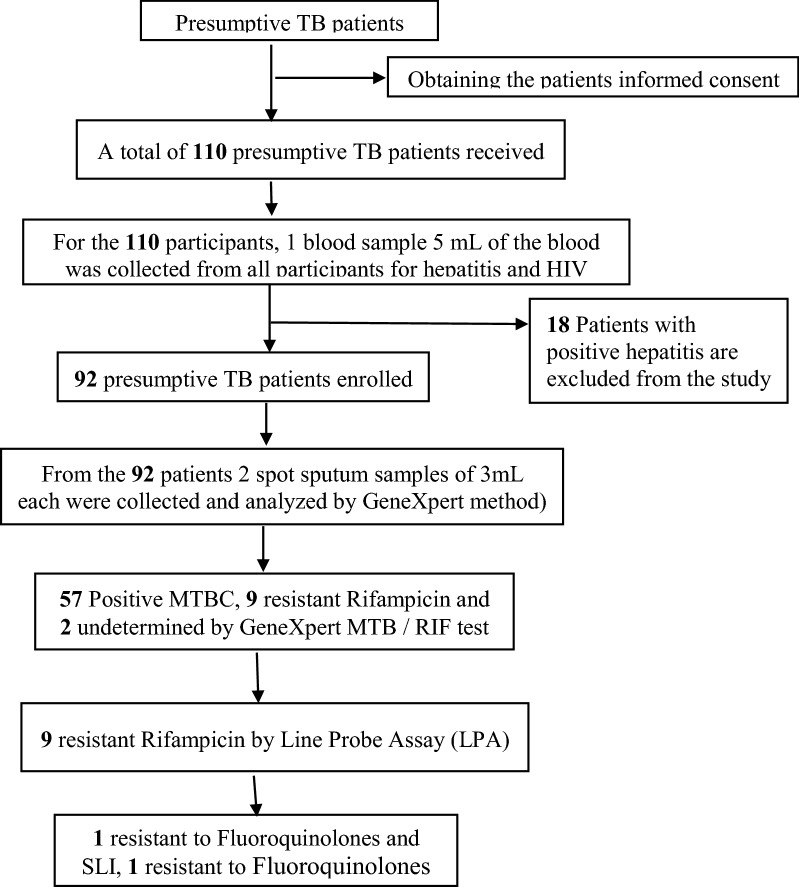
Flowchart of patient recruitment at Makelekele hospital and laboratory methods used

The study design is summarized on Fig. 1. Briefly, after signing the informed consent, the socio-demographic data were collected by the study physician during the clinical examination. A total volume of 5 mL of the blood was collected from all participants for hepatitis and HIV testing (Pre-counseling was done before the HIV test).

Two sputum specimens of 3 mL each were collected from enrolled study participants in accordance with the guidelines of the national tuberculosis control program [15]. The first (spot) sputum specimen was collected at Reference Hospital of Makelekele on the first day. The second (morning specimen) was collected at home on the second day and delivered the same day to the mycobacteria laboratory at Reference Hospital of Makelekele.

The sputum and blood samples were transported at 4–8° C at the Mycobacterium TB laboratory (MTBL) of the Centre de Recherches des Maladies Infectieuses—Christophe Mérieux (CeRMI-CM), Republic of Congo. On receipt of the samples at the MTBL Blood samples were tested for hepatitis with the Hepatitis B rapid test and the Promed Gold Hepatitis C test, as well as for HIV with the Determine HIV 1/2 rapid test (Alere GmbH, Cologne, Germany) and the enzyme-linked immunosorbent assay (ELISA, Vironostika®HIV-1 Plus O Microelisa System, United Kingdom). Sputum samples were decontaminated using the BD BBL® MycoPrep™ Specimen Digestion/Decontamination Kit (Becton Dickinson) following the manufacturer's instructions. The decontaminated samples were stored at -80° C until further analyses.

Detection of *Mycobacterium tuberculosis* complex and rifampicin resistance in the sputum was using the Xpert® MTB/RIF test (Cepheid, Sunnyvale, CA, USA). All rifampicin-resistant samples were subjected to Line probe Assay test (LPA: Geno Type® MTB DR sl assay; Hain Life Science, Gmbh, Germany) to detect resistance to fluoroquinolones and second-line injectable drugs (Amikacin, Kanamycin and Capreomycin).

### GenoType MTBDR sl

From the decontaminated sputum samples, we extracted the DNA by thermal lysis and sonication as described below. Briefly, 500 μL of decontaminated sputum was centrifuged at 10,000×g at 4 °C for 15 min. The supernatant was discarded, the pellet was resuspended in 100 μl of sterile distilled water and the mycobacteria were lysed by incubation at 95 °C for 20 min and the sonication for 15 min, then centrifuged at 13,000 × g for 5 min. The supernatant was collected and stored at − 20 °C [16].

With the extracted DNA, amplification and hybridization were performed with the reagent GenoType MTBDRsl VER2.0 (Hain Lifescience, Nehren, Germany) following the manufacturer's instructions.

### Statistical analysis

The data were analyzed using SPSS Statistical Software version 24(IBM Corp, Armonk, NY). Socio-demographic data, TB risk Behaviours and clinical information were associated to the positivity of GeneXpert method by logistic regression model and their Odd Ratio with the confidence interval at 95% was determinate. The Mann–Whitney test and Kruskal–Wallis test were used to see the dependence of the Rifampicin Susceptibility and the factors. Differences were considered statistically significant when the p-value was < 0.05.

## Results

### Socio-demographic characteristics of the participants

From October 2018 to October 2019, 110 suspected MTB patients were screened at Reference hospital of Makelekele in Brazzaville for TB. Based on clinical examination and inclusion criteria, 92 patients were included in the study, 18 were excluded from the study because of hepatitis B and C positivity (Fig. 1) or manifestation of extrapulmonary tuberculosis.

This total of 92 patients consisted of 47 (51.1%) females and 45 (48.9%) males. The average age was 38.2 (± 15.1) years. However, most were 18–44 51(55.4%), followed by ≥ 45 years 33(35.9%) and ≤ 17 years 8(8.7%) (Table 1).

**Table 1:** Detection of *Mycombacterium tuberculosis* Complex using GeneXpert according the sociodemographic, risk behaviours, co-morbidity and clinical characteristics

Characteristic	All patient (%)	Number of positive MTBC (%)	Crude Odd Ratio (CI.95%)	*P*. value
	N = 92	N = 57		
Sociodemographic				
Age group (years)				
< 18	8 (8.7)	07 (87.5)	1	0.259
18–44	51 (55.4)	34 (66.7)	0.28 (0.03–2.51)	0.071
≥ 45	33 (35.9)	16 (48.5)	0.13 (0.01–1.21)	
Gender				
Female	47 (51.1)	30 (63.8)	1	0.705
Male	45 (48.9)	27 (60.0)	0.85 (0.37–7.97)	
Rain season	57 (62.0)	31 (54.4)	1	
Dry season	35 (38.0)	26 (74.3)	2.42 (0.96–6.09)	0.056
TB risk behaviours				
Alcoholic	43 (46.7)	27 (62.8)	1.07 (0.45–2.48)	0.878
Smoking	20 (21.7)	13 (65.0)	1.18 (0.42–3.32)	0.753
Cannabis	05 (05.4)	04 (80.0)	2.57 (0.27–23.9)	0.395
Co-morbidity with HIV				
No	70 (76.1)	50 (71.4)	1	0.002
Yes	22 (23.9)	7 (31.8)	0.19 (0.07–0.53)	
Clinical				
Chronic cough	66 (71.7)	44 (66.7)	2 (0.79–5.03)	0.140
Acute cough	28 (30.4)	13 (46.4)	0.39 (0.15–0.98)	0.042
Fever	72 (78.3)	45 (62.5)	1.11 (0.40–3.06)	0.839
Anaemia	10 (10.9)	04 (40.0)	0.36 (0.09–1.40)	0.132
Neurological signs	04 (04.3)	03 (75.5)	1.89 (0.19–18.9)	0.508
Night sweat	15 (16.3)	18 (66.7)	1.28 (0.39–4.10)	0.683
Physical asthenia	27 (29.3)	10 (66.7)	1.33 (0.52–3.42)	0.549
Anorexia	19 (20.7)	12 (63.2)	1.07 (0.37–3.03)	0.904
Weight loss	82 (89.1)	54 (65.9)	4.5 (1.08–18.76)	0.039

### Clinical signs, co-infection and associated risk factors of the participants

Of the 92 participants in the study, 66 (71.7%) had a chronic cough, 28(30.4%) had an acute cough, 72 (78.3%) had a fever, 82 (89.1%) had a weight loss, 27 (29.3%) had a physical asthenia and 19 (20.7%) had an anorexia. The HIV positivity rate was 22(23.9%). Of all participants, 43(46.7%) accepted to be alcoholic consumer, 20 (21.7%) recognized to be smoker and 5 (5.4%) recognized to be cannabis consumer (Table 1).

### Proportion of *Mycobacterium tuberculosis* complex (MTBC) positivity and associated characteristics

Of the 92 participants enrolled in the present study, 57 (62%) were found to be positive for MTBC by the Xpert MTB/RIF test. The association between potential exposure variables and the participants' MTBC positivity was analyzed and presented in Table 1. There was a significant association between the participants' positivity to the MTB complex and HIV positivity (95% CI = 0.07–0.53, P = 0.002), as well as an association with acute cough (95% CI = 0.15–0.98, P = 0. 042). However, there was no significant association between the other variables and positivity to the MTB complex.

### Rifampicin sensitivity profile

Of the 57/92 CMTB-positive participants, 2/92 (2%) were indeterminate and so removed from the analysis, 9/55 (16.4%) were resistant to rifampicin, and 46 were rifampicin-sensitive (Table 2).

**Table 2:** The sociodemographic, risk behaviors, co-morbidity and clinical characteristics associated with RR-TB status among participants positive for *Mycobacterium tuberculosis* complex by GeneXpert

Characteristic	Rifampicin Susceptible (%)	Rifampicin Indeterminate (%)	Rifampicin Resistant (%)	*P.* value
	N = 46	N = 2	N = 9	
Sociodemographic				
Age group (years)				
< 18	4 (8.7)	1 (50.0)	2 (22.2)	0.323
18–44	30 (65.2)	1 (50.0)	3 (33.3)	
≥ 45	12 (26.1)	0	4 (44.5)	
Gender				
Female	22 (47.8)	2 (100.0)	6 (66.7)	0.156
Male	24 (52.2)	0	3 (33.3)	
Rain season	26 (56.5)	2 (100.0)	3 (33.3)	0.191
Dry season	20 (43.5)	0	6 (66.7)	
TB risk behaviours				
Alcoholic	24 (52.2)	0	3 (33.3)	0.236
Smoking	12 (26.1)	0	1 (11.1)	0.462
Cannabis	3 (6.5)	0	1 (11.1)	0.822
Comorbidity with HIV				
No	43 (93.5)	2 (100.0)	5 (55.6)	0.006
Yes	3 (6.5)	0	4 (44.4)	
Clinical				
Chronic cough	38 (82.6)	0	6 (66.7)	0.019
Acute cough	8 (17.4)	2 (100.0)	3 (33.3)	0.019
Fever	35 (76.1)	2 (100.0)	8 (88.9)	0.529
Anaemia	1 (2.2)	1 (50.0)	2 (22.2)	0.006
Neurological signs	3 (6.5)	0	0	0.689
Night sweat	8 (17.4)	1 (50.0)	1 (11.1)	0.431
Physical asthenia	14 (30.4)	2 (100.0)	2 (22.2)	0.098
Anorexia	11 (23.9)	1 (50.0)	0	0.168
Weight loss	44 (95.7)	2 (100.0)	8 (88.9)	0.673

The association between the potential exposure variables and the participants' rifampicin-resistant tuberculosis (RR-TB) was analyzed and presented in Table 2. Rifampicin-resistant tuberculosis was statistically significant in HIV-positive participants (P = 0.006), there was a significant association between rifampicin-resistant tuberculosis and chronic cough (P = 0.019), also a significant association with acute cough (P = 0.019), and a significant association with anaemia (P = 006) and physical asthemia (P = 0.098). However, there was no significant association between the other variables and RR-TB.

### Sensitivity profile for second-line drugs

For the 9 participants resistant to rifampicin, we found that 1/9 (11.1%) was resistant to fluoroquinolones and 1/9 (11.1%) was resistant to both fluoroquinolones and injectable second line drugs. The remaining 7 rifampicin-resistant participants were sensitive to second line drugs.

## Discussion

The present study showed that the prevalence of rifampicin-resistant tuberculosis (RR-TB) among the 92 participants suspected of having tuberculosis and who had never received treatment was 9.8% (9/92) and importantly 2.2% were pre-XDR/XDR. The data reported in the study area do not allow a more precise analysis of the exact causes of this increase (type of exposure, contact persons). However, the detection of the relatively high rate of RR-TB resistance in the study population could be due to the late diagnosis (patients with resistant strains are diagnosed late and often at the advanced stage of the disease) which would be responsible for the spread of resistant strains in the community. The lack of access to in vitro culture facilities and limited availability of rapid molecular diagnostic tools in some hospitals means that the delay in diagnosing resistance is often long [17].

According to the national TB control program in 2013, only patients in treatment failure with positive microscopy after five months of treatment should benefit of GeneXpert investigation. The main reason, is the limited financial resources leading to shortages in the purchase of cartridges [14]. However, since 2019, the National Tuberculosis Program has implemented a new algorithm that consists of molecular testing as the initial diagnostic test for the detection of tuberculosis to anyone with symptoms of TB [18]. This algorithm is proposed to serve the goals of the "End TB" strategy, the rapid diagnostic tests recommended by WHO. The molecular tool implemented is the GeneXpert, which makes the accurate and rapid detection of tuberculosis (TB) and rifampicin resistance, which allows for improved patient management.

According to the WHO, the estimate of MDR-TB/MDR-TB at the national level was 2.4% in new patients. The discrepancy with the estimate reported in this study could be explained by the small study sample size, and the difficulty to extrapolate this result to the national level. However, in the absence of national data, this study provides an initial assessment of the situation in the Republic of Congo and should be considered as such.

The prevalence of RR-TB found in the present work is lower than that reported in the study conducted in Gabon, in a sample of 124 patients (new and failed treatment), a prevalence of RDR-TB of 17% was noted [19]. Indeed, an earlier study conducted in the Republic of Congo at the Tuberculosis Center reported a prevalence of 18% in a sample of 111 patients in treatment failure [14]. Importantly, our study was conducted only in new patients, in contrast to this study, and this is a cause for concern as it indicates increased transmission of drug resistant forms of *Mycobacterium tuberculosis*.

It should be noted that, in most cases, it is possible to cure rifampicin-resistant tuberculosis, although second-line treatment is long and requires strict adherence to a treatment regimen with support and supervision of the patient during treatment [20]. First- and second-line treatment of tuberculosis is provided free of charge by the National Tuberculosis Control Program in the Republic of Congo [21].

Our study also showed that HIV status (p = 0.006) was the only significant factor (p = 0.005) associated with rifampicin-resistant tuberculosis; other factors such as alcohol, tobacco, and cannabis use were not significant. Thus, there is a relationship between rifampicin-resistant tuberculosis and HIV status. This could be explained by the fact that tuberculosis is the most common opportunistic disease in HIV-infected patients. This finding has also been made by several authors [22–24].

Clinical signs such as chronic cough (p = 0.019), acute cough (p = 0.019), anemia (p = 0.06) and physical asthenia (p = 0.098) were statistically significant (p = 0.005). The clinical signs of susceptible tuberculosis are the same as those of resistant tuberculosis according to the WHO. Other authors have also pointed out the presence of these clinical signs in relation to rifampicin-resistant tuberculosis [21, 25].

In this study, genotypic testing for second-line drug susceptibility was performed on the 9 participants detected with rifampicin-resistant tuberculosis using the GenoType MTBDR sl test. This test recorded 1 participant who was resistant to fluoroquinolones and also 1 participant who was resistant to both fluoroquinolones and second line injectable drugs. The proportion of this form of extensively drug-resistant tuberculosis in our study population is of concern, requiring significant community action to prevent the spread of resistant strains. These data suggest that efforts should be made to ensure that all patients diagnosed with XDR-TB undergo sensitivity testing with fluoroquinolones and second-line injectables in order to initiate early and effective treatment. A study conducted in the Republic of Congo reported that in a sample of 13 previously treated patients, a proportion of 3 patients were resistant to fluoroquinolones and 1 patient was resistant to fluoroquinolones and second-line injectable drugs [26]. The particularity of our study is that we detected this form of resistance in untreated patients, whereas in the Okemba-Okambi et al. study, this form of resistance was detected in previously treated patients.

It is important to make the right diagnosis at an early stage in order to initiate effective treatment as early as possible. It is possible to cure XDR-TB, but with the drugs currently available, the probability of cure remains low. Cure depends on the extent of drug resistance, the severity of the disease and the state of the immune system [1].

In order to be in line with the sustainable development goals, the national tuberculosis control program of the Republic of Congo is in the process of setting up a functional and quality national reference laboratory for mycobacteria to ensure the culture of mycobacteria, anti-TB (DST) susceptibility testing to confirm multidrug resistance prior to the initiation of treatment for MDR-TB patients and second-line anti-TB testing to exclude the existence of pre-XDR/XDR-TB strains with the aim of interrupting the chain of transmission and the resurgence of multi/extra-drug resistant TB.

Nevertheless, the national TB control program in the Republic of Congo provides first and second line anti-tuberculosis treatment for MDR-TB to all patients detected by molecular tools. Treatment is often long and requires good compliance to ensure successful care management. A standardized treatment protocol based on the combination of anti-tuberculosis drugs during two phases: the first intense phase lasts 6 months and the second continuation phase lasts 3 to 20 months is recommend by the national TB control program [26].

This study is the first to show a high prevalence of RR-TB and the presence of XDR-TB in patients suspected of having tuberculosis and who had never received treatment, in the Republic of Congo. These findings provide solid evidence regarding TB case management and advocate for a well equipped national mycobacteria reference laboratory.

Obviously, it would be necessary to extend this investigation to other hospitals not only in Brazzaville but also in other cities in order to have a better understanding of the current status of the epidemiological profile of MTB resistance. It would also be important to strengthen surveillance systems for tracking resistance to first- and second-line anti-tuberculosis drugs.

Unfortunately, timely drug susceptibility testing (DST) was not performed in this study using other first-line anti-tuberculosis drugs to determine the presence of polyresistant TB in these Congolese isolates. In addition, the small sample size did not allow us to make any comparison with other studies.

## Conclusion

This study reported a high rate of rifampicin resistance and the presence of extensively drug-resistant tuberculosis in new diagnosed Congolese patients. This study highlights the need for further studies of TB drug resistance using fast and reliable detection of resistant TB-cases in the country.

## Data Availability

Not applicable.

## Abbreviations

MDR-TB: Multidrug- Resistant-Tuberculosis
TB: Tuberculosis
XDR-TB: Extensively drug-resistant TB
WHO: World Health Organization
DST: Drug susceptibility testing
LPA: Line probe Assays
RR-TB: Rifampicin-resistant tuberculosis
